# Revised relationships of the endemic New Zealand subfamily Pronkiinae stat. nov. (Hymenoptera, Braconidae)

**DOI:** 10.3897/zookeys.1294.210518

**Published:** 2026-10-13

**Authors:** Donald L. J. Quicke, Darren F. Ward, Michael J. Sharkey, Buntika A. Butcher

**Affiliations:** 1 Center of Excellence in Integrative Insect Ecology, Department of Biology, Faculty of Science, Chulalongkorn University, Phayathai Road, Pathumwan, Bangkok 10330, Thailand Center of Excellence in Integrative Insect Ecology, Department of Biology, Faculty of Science, Chulalongkorn University Bangkok Thailand; 2 New Zealand Arthropod Collection, Bioeconomy Science Institute, Auckland, New Zealand New Zealand Arthropod Collection, Bioeconomy Science Institute Auckland New Zealand; 3 The Hymenoptera Institute, 3028 Arrowhead Drive, Lexington, Kentucky 40503, USA The Hymenoptera Institute Kentucky United States of America

**Keywords:** COI, 28S, 18S, 16S, braconid wasp, elongation factor-1 alpha, heritage genes, sequence assembly, sigalphoid, taxonomy

## Abstract

The endemic New Zealand braconid wasp tribe Pronkiini (type and only genus *Pronkia* van Achterberg, 1990) has, until now, been placed in the subfamily Meteorideinae. We obtained the first molecular data for *Pronkia* comprising sequences for five heritage gene fragments (the barcoding region of COI, the nuclear protein-coding elongation factor-1 alpha gene, and three ribosomal genes, 28S, 18S, and 16S) and analysed them together with data from a wide range of other non-cyclostome braconid subfamilies. None of our analyses support the inclusion of *Pronkia* within Meteorideinae; instead, *Pronkia* was placed in the strongly supported sigalphoid clade when each of the genes was analysed separately, as well as in the analysis of the combined dataset. In the latter, it was recovered as a sister group to Sigalphinae, but with very low support, essentially in a trichotomy with each of the three sigalphoid subfamilies monophyletic with both 100% UFB and SH-aLTR support. We therefore elevate Pronkiini to subfamily status, Pronkiinae**stat. nov**., and provide a diagnosis of the new subfamily, along with a photographic portfolio of representative species. Morphological characters relevant to Pronkiinae**stat. nov**. relationships are discussed.

## Introduction

The parasitoid wasp fauna of New Zealand, although still poorly known, includes many endemic genera and several basal taxa of various braconid subfamilies (Fernandez-Triana et al. 2011; Quicke et al. 2019a, 2019b, 2019c, 2020a; Ward 2025). The cosmopolitan and highly diverse subfamily Braconidae is currently represented in New Zealand by just 99 indigenous species in 52 genera (Ward 2025). Sixteen of these genera are known only from New Zealand, a remarkably high degree of endemism of 30% (Ward 2025). One of these genera, *Pronkia* van Achterberg, 1990, is currently placed in Meteorideinae together with its only other genus, *Meteoridea* Ashmead, 1900, but in the separate tribe Pronkiini (van Achterberg 1990). *Pronkia* includes a single described species, but DNA barcoding suggests several undescribed species (Ward 2024). Van Achterberg (1990), in his original description, expressed some uncertainty about this placement and presented considerable discussion about its affiliations, particularly debating between Meteorideinae and Agathidinae, and finally opting for the former. However, the phylogenetic relationships of *Pronkia* have never been formally assessed.

Here we present a molecular phylogeny based on five heritage gene fragments – COI, 28S, 18S, 16S and elongation factor-1 alpha (EF1-α) – for a broad range of non-cyclostome braconid representatives. We found that *Pronkia* belongs to neither Meteorideinae nor Agathidinae, although it is well-supported as a member of the Agathidinae+Sigalphinae clade, often referred to informally as the sigalphoid group of subfamilies (Sharanowski et al. 2011). Accordingly, we raise the status of Pronkiini to subfamily rank, hence Pronkiinae stat. nov. In addition, we provide a diagnosis of the new subfamily-level taxon and illustrate selected species photographically for the first time.

## Materials and methods

### Taxon sampling

We assembled a data set comprising DNA sequences of representative members of almost all non-cyclostome braconid subfamilies (viz., Acampsohelconinae, Afrocampsinae, Agathidinae, Amicrocentrinae, Brachistinae, Cardiochilinae, Cenocoeliinae, Charmontiinae, Euphorinae, Homolobinae, Ichneutinae, Macrocentrinae, Meteorideinae, Microtypinae, Orgilinae, Proteropiinae, Sigalphinae and Xiphozelinae) and for three species of *Pronkia*. Taxon sampling emphasised the sigalphoid lineage as well as data from multiple species of *Meteoridea*. Of the microgastroid lineage, we included only a small number of representatives, as their monophyly is well documented (Whitfield and Mason 1994; Dowton and Austin 1998; Sharanowski et al. 2011; Jasso-Martínez et al. 2022a, 2022b).

Since the analyses rely heavily on legacy genes that were often sequenced for different species in various published investigations, we pooled some data for different species within the same genus (Whitfield and Lockhart 2007). Details of the sources and identities of all the sequences analysed are given in Suppl. material 1.

### Molecular data

All taxa were represented by the barcoding region of the mitochondrial cytochrome oxidase *c* subunit 1 (COI). Many additional taxa were represented by the D2-D3 expansion region of the nuclear 28S rDNA (28S) gene, up to 1118 base pairs, of which 635 were considered reliably alignable, the nuclear 18S rDNA (18S) gene, up to 1450 base pairs of which 1346 were alignable, the mitochondrial 16S rDNA (16S) gene between the core I region and five bases after H2675, a length comprising approximately 1140 bases of which 534 were considered reliably alignable, and by the F2 copy of the nuclear elongation factor-1 α (EF1-α) gene (418 base pairs) (Belshaw and Quicke 1997; Quicke et al. 2020b, 2020c). Some COI, 28S, 16S and EF1-α sequences were newly generated by the Centre for Biodiversity Genomics, University of Guelph, or by the Bioeconomy Science Institute in New Zealand based on standard protocols as described in Hebert et al. (2003), Hajibabaei et al. (2005), Park et al. (2010) and Wilson (2012). Most recently, some EF1-α sequences were obtained using Oxford Nanopore technology (Kolter and Hebert 2025; Hebert et al. 2025). Additionally, we manually assembled COI, 16S, 18S and 28S sequences from published secondary products of UCE libraries in GenBank and extracted some COI and 16S sequences from published mitogenomes (Wei et al. 2010; Fujie et al. 2019; Jasso-Martínez et al. 2022a). Various combinations of these gene fragments have been widely employed in braconid phylogenetics (e.g., Dowton and Austin 1998; Dowton et al. 2002; Shi et al. 2005; Kittel et al. 2016) and for discussion of the relative utilities, see Dowton and Austin (1994, 2001), Sharanowski et al. (2011), and Quicke et al. (2021).

### Sequence alignment

Alignment of the COI and EF1-α gene fragments was straightforward, as there were no indels (Suppl. materials 4, 5). The 16S rRNA gene regions were aligned according to the secondary structure model of Wu et al. (2014) with additional reference to Buckley et al. (2000) for helix nomenclature. The length-variable 28S sequences were aligned according to the secondary structure model of Gillespie et al. (2005) as in other studies (Butcher et al. 2014; Quicke et al. 2020b, 2020c; Quicke et al. 2024). For the 28S gene, alignment of the sigalphoid sequences (including those of *Pronkia*) with those of the other taxa was complicated because the former have highly divergent sequences and sequence lengths. In particular, the sequence of the D3-1 to D3-1’ helix and loop of *Pronkia* bore no resemblance to that of any other taxon but was alignable within the three included species of *Pronkia*, so that region was offset from the sequences of the other taxa.

The three aligned ribosomal gene files with their secondary structure interpretations and paired, unpaired and excluded bases, are given in Suppl. materials 6–8. For simplicity, all pairing bases were placed in one contiguous block for each ribosomal gene, followed by the respective unpairing bases. The final concatenated data set analysed is provided in Suppl. material 9. All DNA alignments are also available on Datadryad.org (see Data Availability Statement), and all data for the total of 205 sequenced *Pronkia* species are available at dx.doi.org/10.5883/DS-PRONKIA (see Data Availability for further details).

The COI and EF1-α sequences were both partitioned according to codon position. Confidently alignable regions of each ribosomal gene sequence were partitioned into pairing (plus bases involved in three-way interactions, i.e., base triplets; Gillespie et al. 2006) and unpairing bases, giving a total of 12 data partitions.

### Phylogenetic analyses

We performed maximum-likelihood analyses with IQ-TREE 3 (version 3.1.1) (Wong et al. 2026) using the relevant partitioned datasets (Chernomor et al. 2016) and the best-fit models determined by ModelFinder (Kalyaanamoorthy et al. 2017) based on the corrected Bayesian Information Criterion (BIC). Branch support was estimated using ultrafast bootstrap (UFB) (1000 replicates) (Minh et al. 2013; Hoang et al. 2018), standard non-parametric bootstrap (BS) (100 replicates) and the Shimodaira and Hasegawa-like approximate likelihood ratio test (Guindon et al. 2010) (SH-aLRT) (1000 replicates). We recommend that a phylogenetic branch be considered reliable if UFB support is ≥ 95% and SH-aLRT ≥ 80% (Guindon et al. 2010; Minh et al. 2013).

Trees were visualised using FigTree v1.4.4 (Rambaut 2018) and rooted on *Meteoridea* Ashmead (Meteorideinae) which was recovered as the sister group to all other non-cyclostome braconids in the UCEs study (Jasso-Martínez et al. 2022b).

### Morphology

General morphological terminology follows van Achterberg (1988) and Sharkey and Wharton (1997), the Hymenoptera Anatomy Ontology Portal (http://portal.hymao.org/projects/32/public/ontology/) and for cuticular sculpture, which follows Harris (1979). Wing venation terminology follows Sharkey and Wharton (1997) (see also Quicke, 2015). Images of *Pronkia* were taken with a Canon EOS RP camera (26 megapixels) using Canon EOS Utility 3.20 and Zerene Stacker [build T2026-05-04-0750] software.

## Results

### Phylogenetic results

**Figure 1.**
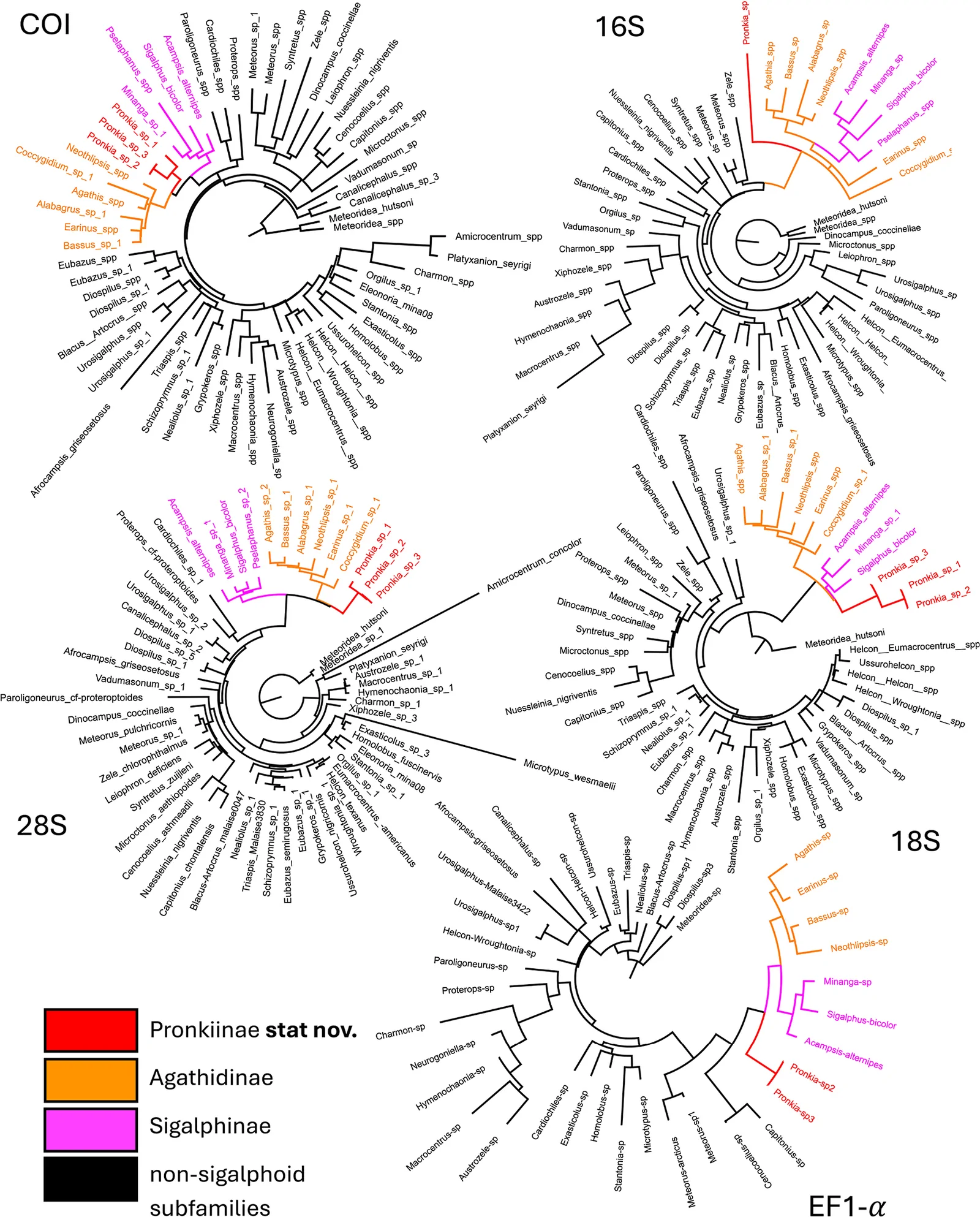
Maximum-likelihood phylogenies based on separate analyses of five single-gene fragments, viz., COI, 28S, 18S, 16S and elongation factor-1 α. Each is rooted with Meteorideinae. Members of the sigalphoid clade, indicated in colour: red = Pronkiinae stat. nov., orange = Agathidinae, magenta = Sigalphinae.

The IQ-TREE3 maximum-likelihood trees obtained for each individual gene are shown in Fig. 1, with members of the three subfamilies comprising the sigalphoid clade indicated in colour.

**Figure 2.**
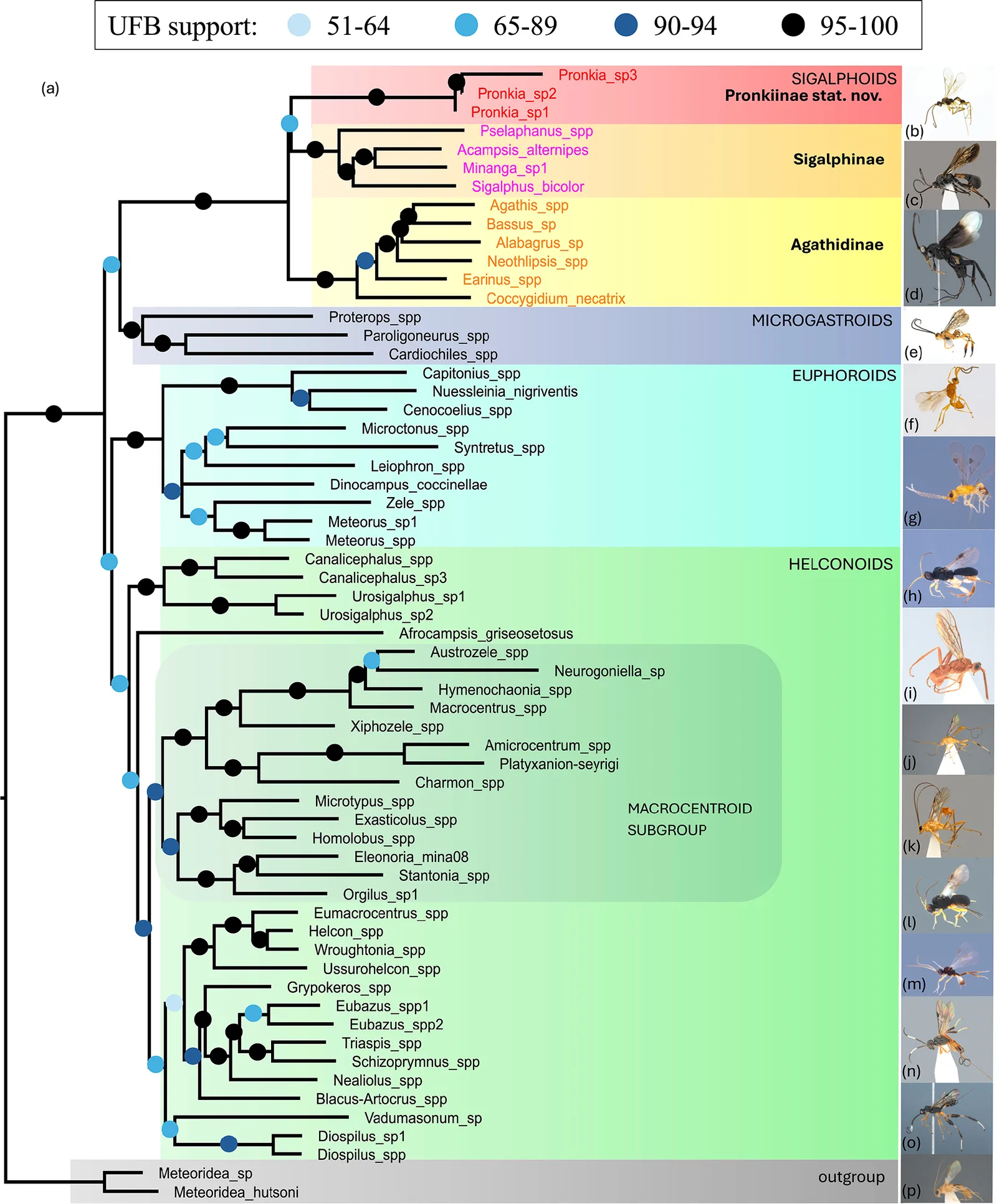
**a**. IQTREE-2 maximum-likelihood phylogeny based on analysis of five concatenated gene fragments with ranges of bootstrap percentage support for nodes indicated; **b**. *Pronkia* sp. (Pronkiinae stat. nov.); **c**. *Sigalphus* sp. (Sigalphinae); **d**. *Biroia fuscicornis* (Agathidinae); **e**. *Hartemita* sp. (Cardiochilinae); **f**. *Canalicephalus* sp. (Afrocampsinae); **g**. *Streblocera* sp., (Euphorinae); **h**. *Cosmophorus* sp. (Euphorinae); **i**. *Homolobus* sp. (Homolobinae); **j**. *Stantonia* sp. (Orgilinae); **k**. *Austrozele* sp. (Macrocentrinae); **l**. *Triaspis* sp. (Brachistinae); **m**, **n**. Helcon (Helconidea) sp. (Helconinae); **o**. *Meteoridea* sp. (Meteorideinae). (All photographs © B. A. Butcher and D. L. J. Quicke, from Butcher and Quicke (2023).

In all of the five individual gene analyses, Pronkiinae formed a monophyletic clade with Agathidinae and Sigalphinae. However, the exact relationships among the sigalphoid subfamilies varied by gene. *Meteoridea* was never recovered near *Pronkia* in analyses of the individual gene datasets (Fig. 1) or the combined dataset (Fig. 2).

In the ML tree from the combined data set (Fig. 2) *Pronkia*+ Agathidinae + Sigalphinae formed a monophyletic group with 100% UFB (Suppl. material 2) and SH-aLTR (Suppl. material 3) support, and each of these three groups was also supported as monophyletic with 100% support with both measures. However, the relationship between the three was not clearly resolved.

## Taxonomy

### Pronkiinae

Taxon classificationAnimaliaHymenopteraBraconidae

van Achterberg, 1990 stat. nov.

A45F29B6-DD58-5685-A8D5-730784FB54F5

#### Type and only known genus.

*Pronkia* van Achterberg, 1990.

#### Diagnosis.

**Figure 3.**
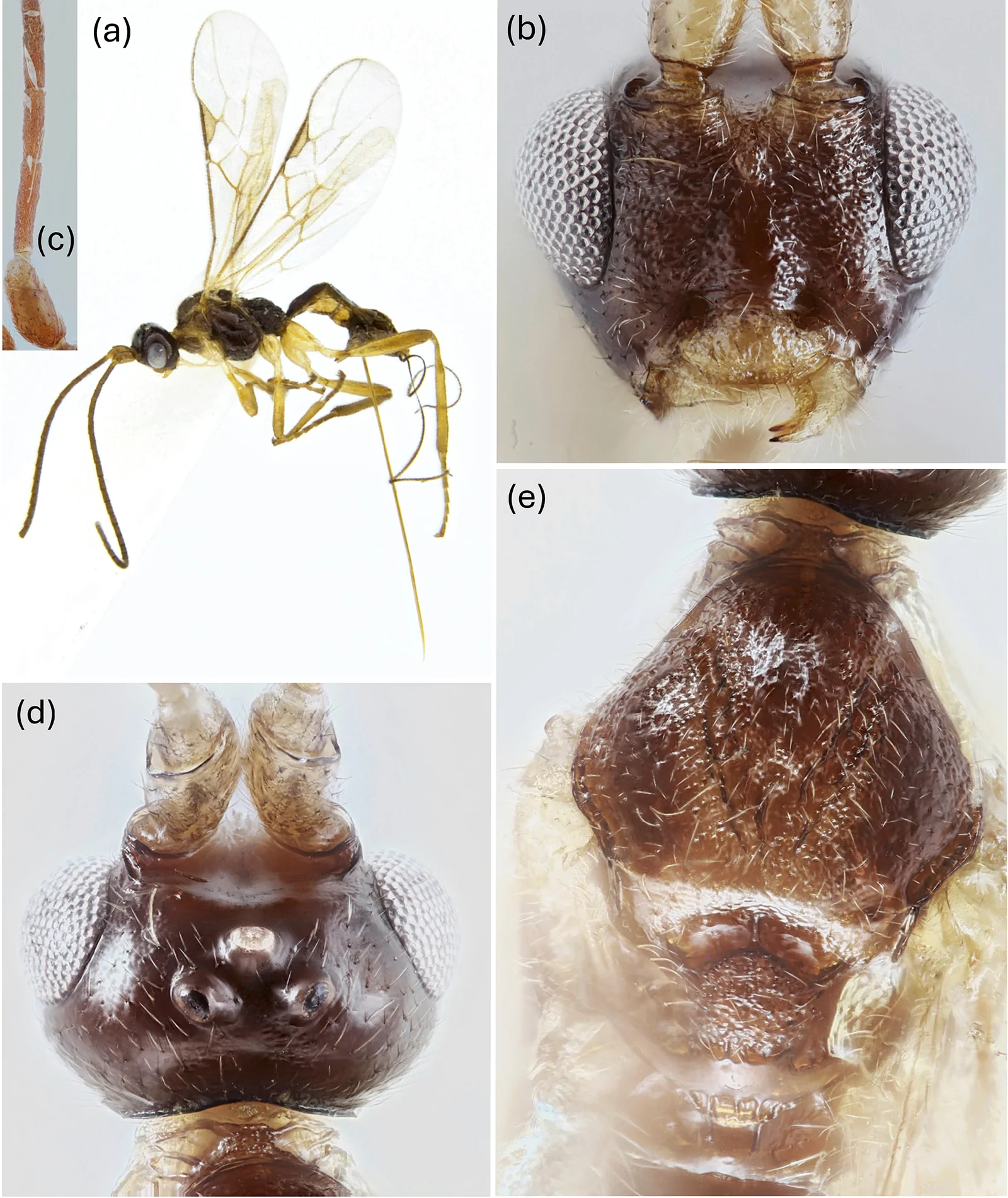
Features of *Pronkia* species. **a**. *Pronkia antefurcalis*, female habitus, lateral view; **b**. *Pronkia* sp., head, anterior view; **c**. Scape, pedicel and basal three flagellomeres; **d**. Head and anterior pronotum, dorsal view; **e**. Mesothorax, dorsal view. Vouchers: (a) NZAC04248716; (b-e) NZAC04106068.

**Figure 4.**
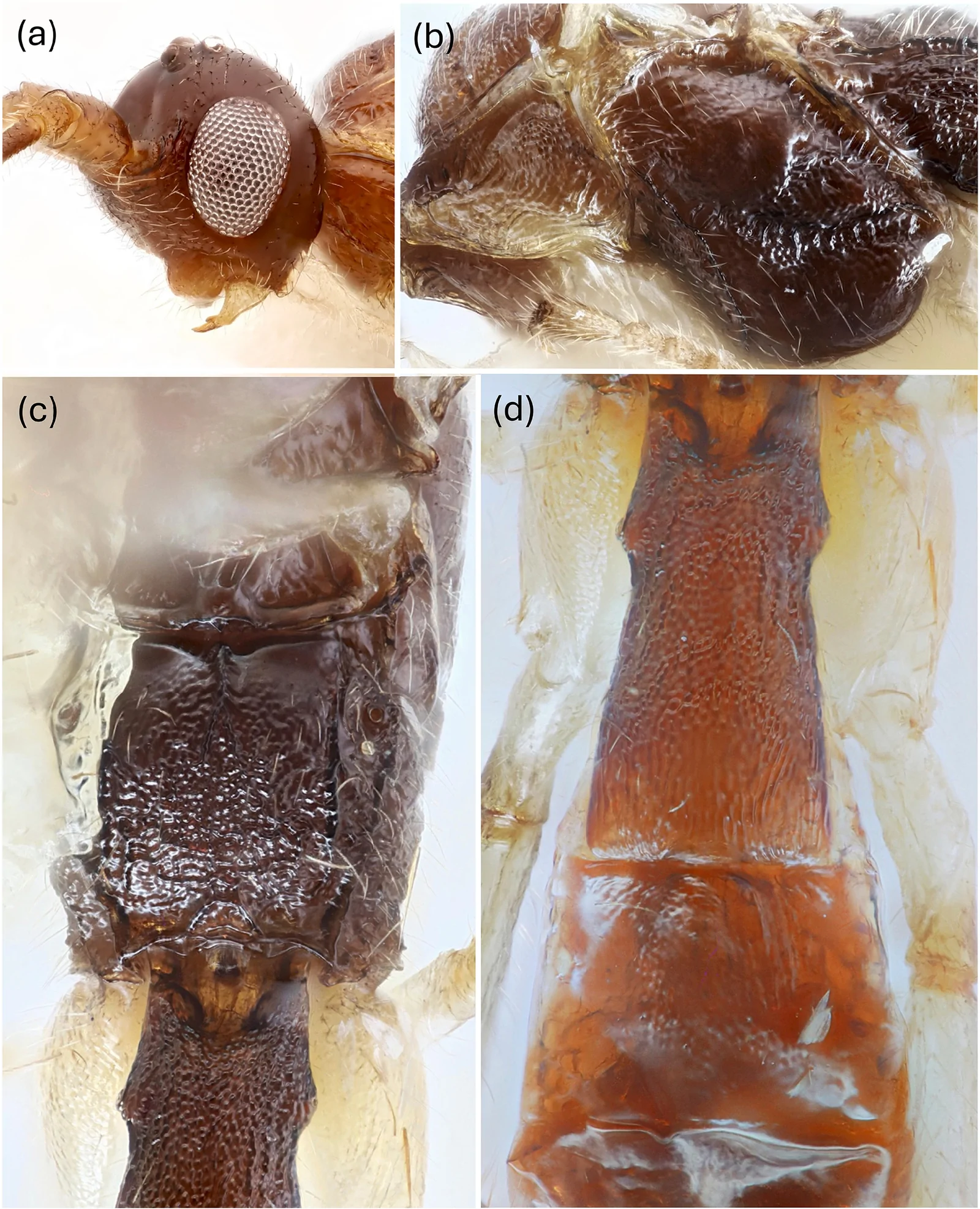
Features of *Pronkia* species. **a**. Head, antero-dorsal oblique view; **b**. Mesosoma, lateral view; **c**. Metanotum, propodeum and anterior metasomal tergite 1, dorsal view; **d**. Metasomal tergites 1–3, dorsal view. Vouchers: (a) NZAC04248716; (b) NZAC04106068; (c, d) NZAC04106607.

**Figure 5.**
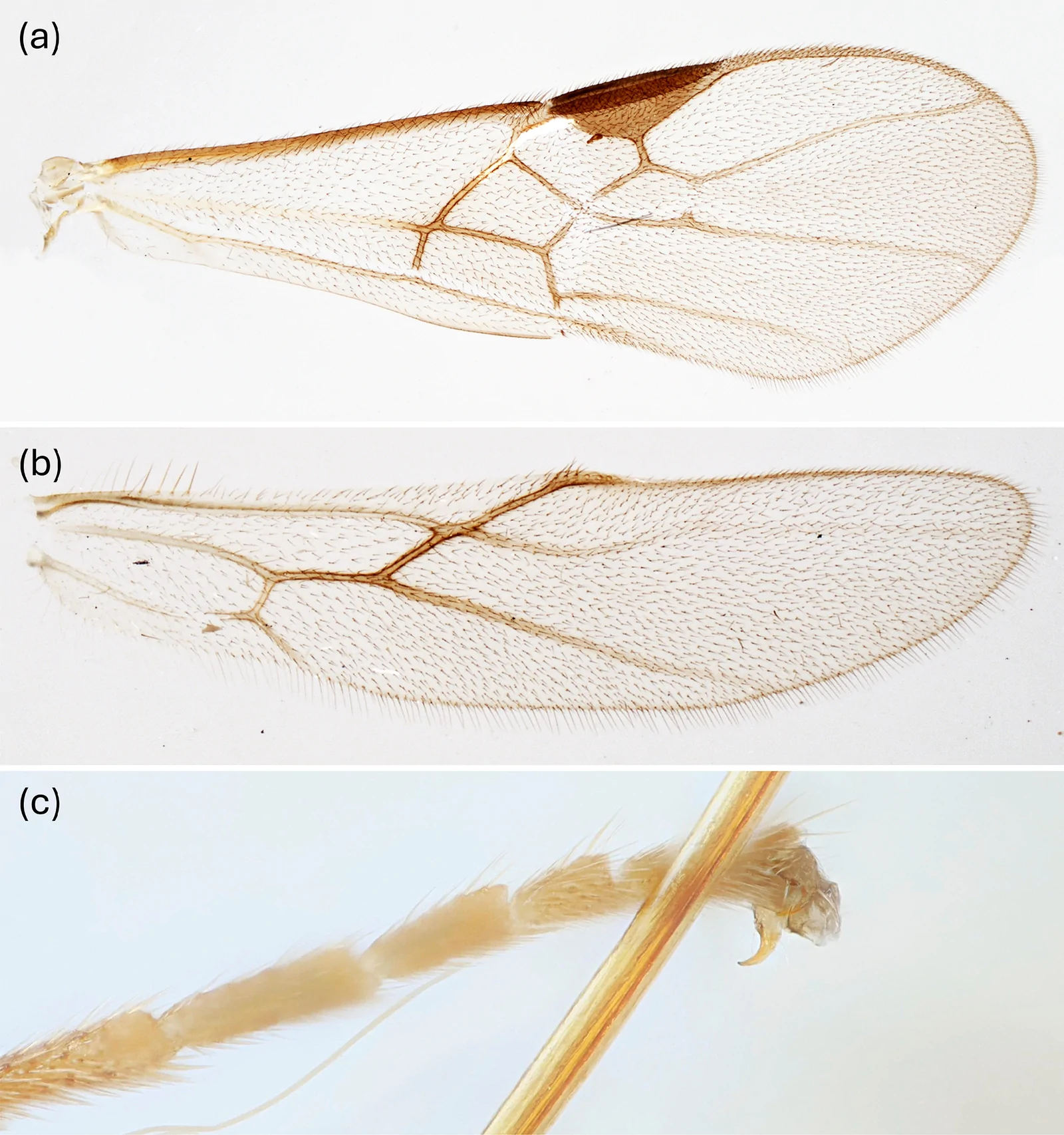
Features of *Pronkia* species. **a**. Fore wing; **b**. Hind wing; **c**. Hind tarsus and claw, inner aspect. Vouchers; (a, b) NZAC02011904; (c) NZAC04106068.

Small wasps with body lengths 2.5–3.5 mm, body largely with granulate and other sculpture, females with well-exserted, long ovipositors approximately as long as fore wing. Non-cyclostome (Figs 3b, 4a). Antenna with 26–29 flagellomeres, all flagellomeres elongate (Fig. 3c). Occipital carina complete (Fig. 3d), joining hyposomal carina far above base of mandible (Fig. 4a). Pronope variable, subpronopes well developed (Fig. 3e). Scutellum with transverse subposterior depression (Fig. 3e). Prepectal carina complete (Fig. 4b). Precoxal sulcus complete and strongly sculptures (Fig. 4b). Pleural suture crenulate (Fig. 4b). Fore wing (Fig. 5a): vein 1RS distinct; marginal cell wide (not short and narrowed as in Agathidinae); 2^nd^ submarginal cell four-sided, vein 3SR 2–3× longer than crossvein r-rs; vein (RS+M)a complete and tubular; vein M+CU largely unsclerotised; vein 1cu-a antefurcal; vein 2cu-a present; distinct trace of vein 1a visible in some specimens. Hind wing (Fig. 5b): vein CUb well developed, tubular basally, arising far distal to the crossvein cu-a bulla; basal hamules (especially thickened setae) arise from tubular part of vein C+SC+R. Hind tibia clavate, indicating presence of well-developed subgenual organ. Claws with small, smoothly rounded basal lobes (Fig. 5c). Metasomal tergite 1 either without dorsal carinae and dorsope (van Achterberg, 1990) or with weak short curved dorsal carinae demarking shallow dorsopes (Fig. 4c, d). Apex of ovipositor smooth, without preapical dorsal notch.

In van Achterberg’s (van Achterberg 1993) illustrated key to the subfamilies of Braconidae, Pronkiinae stat. nov. will run to Meteorideinae at couplet 11. It may be distinguished from that subfamily by the features given in van Achterberg (1990): (1) presence of well-developed fore wing vein 1RS (Fig. 5a); (2) largely unsclerotised fore wing vein M+CU (Fig. 5a); (3) fore wing vein 1cu-a antefurcal (Fig. 5a); and female with long, exserted ovipositor (Fig. 3a).

The 202 barcodes of *Pronkia* currently on the BOLD database (accessed 7 Feb. 2026) collectively represent nine BINs (BOLD:AEG6525, BOLD:AES5972, BOLD:AEG3766, BOLD:ADO3682, BOLD:ADO1200, BOLD:ADO0706, BOLD:ACP2920, BOLD:ABX8289 and BOLD:AAV1740) suggesting that the genus is represented by nine or more species.

## Discussion

Morphological data provide an ambiguous indication of the relationships of *Pronkia*. Among non-cyclostome Braconidae, hind wing vein 2-CU is found in several subfamilies (Acampsohelconinae, Afrocampsinae, Agathidinae, Meteorideinae and Trachypetinae), and among the non-cyclostomes (probably a grade taxon), it is found only in the monotypic Chilean Apozyginae as well as in many Cretaceous and a few Eocene fossils, though not in any other extant taxa. We therefore suspect that it may have appeared secondarily among at least some of the above groups, since those subfamilies possessing vein 2CU are not all clustered together as a clade or grade in the recent phylogenomics tree of Braconidae (Jasso-Martínez et al. 2022b).

Analysis of the concatenated molecular dataset recovered relationships between non-cyclostome braconid subfamilies that are broadly similar to those obtained by Sharanowski et al. (2011) based on multiple nuclear genes and by Jasso-Martínez et al. (2022b). The main exception is the two tribes of Afrocampsinae (formerly part of Acampsohelconinae, see Belokobylskij et al. 2025). Chen and van Achterberg (2019) expressed uncertainty about the major subgroup to which Meteorideinae belonged, suggesting tentatively that they might be sigalphoids. They were 50% correct, as we show here that *Pronkia* is indeed a sigalphoid but not related to *Meteoridea*, which is far more basal among the non-cyclostomes (Jasso-Martínez et al. 2022b).

Despite their shared possession of hind wing vein 2CU, a close relationship between Sigalphinae and Agathidinae and their separation from other non-cyclostome subfamilies was not widely accepted until formal phylogenetic analyses, especially those with molecular data. Van Achterberg (1984), in his informal morphological cladogram, placed both in a clade broadly corresponding to the current concept of ‘helconoids’, though not as sister taxa. Additional evidence that Sigalphinae and Agathidinae were closely related was provided by Rahman et al. (1998), who showed that members of these subfamilies (including *Pselaphanus*, then included in Pselaphaninae) had a distinctive and putatively synapomorphic form of microsculpture on the egg canal wall of the ovipositor.

Monophyly of the sigalphoid lineage (Agathidinae + Sigalphinae) is strongly supported molecular (Dowton et al. 1998; Belshaw and Quicke 2002; Dowton et al. 2002; Pitz et al. 2007; Sharanowski et al. 2011; Jasso-Martínez et al. 2022a, 2022b, 2023), and now also including Pronkiinae stat. nov., although the relationships of sigalphoids to other braconid subfamilies are less clear (Sharkey 1992). A consensus of recent molecular studies is that they probably are a sister group to the microgastroid clade (Sharanowski et al. 2011; Jasso-Martínez et al. 2022a, 2022b), which almost exclusively share the same basic biology, although some Ichneutinae attack sawfly larvae. Quicke (2015) summarises the oviposition and early developmental biology of agathidines and sigalphines, all of which are exclusively koinobiont endoparasitoids of Lepidoptera caterpillars (Shaw and Huddleston 1991; Chen and van Achterberg 2019; Shaw and Quicke 2000). Further, all sigalphoids have ovipositors that lack the preapical dorsal notch typical of many non-cyclostome braconids, ophioniform ichneumonids and Figitidae (Cynipoidea) (Buffington 2007; Boring et al. 2009; Quicke 2015). In sigalphoids, the ovipositor penetrates the host caterpillar and (with one known exception) places a hydropic egg either inside or attached to the ventral nerve cord or cerebral ganglion, a strategy that prevents or restricts encapsulation by host haemocytes through the early stages of development. The egg swells and hatches into a polypodiform 1^st^ instar larva (Clausen 1940; Quednau 1970; Shaw and Quicke 2000), a larval form that is extremely uncommon among Ichneumonoidea. Given the similarity of the ovipositor of *Pronkia* and its close relationship with the two other sigalphoid subfamilies, it is tempting to speculate that it will also be found to attack concealed Lepidoptera caterpillars and oviposit within them, placing its egg in some protective tissue of the host, such as the nervous system, where host haemocytes are absent.

## Supplementary Material

XML Treatment for Pronkiinae

## Additional information

### Artificial Intelligence (AI) use

The authors accept full responsibility for the content of the manuscript, including the disclosure of any use of AI.

No AI tools were used in the preparation of this manuscript.

### Author ORCIDs

D.L.J. Quicke https://orcid.org/0000-0003-4471-6775

D.F. Ward https://orcid.org/0000-0002-4509-8553

M.J. Sharkey https://orcid.org/0000-0001-6201-7340

B.A. Butcher https://orcid.org/0000-0002-0541-0709

### Data availability

The data that support the findings of this study are openly available at https://chula-my.sharepoint.com/:f:/g/personal/buntika_a_chula_ac_th/IgD_1AWuQ0fnTY7imCVXqLtLAWyq14BVyojN1m3mcskfVuQ?e=Y54Zc4. Additionally, a data set with all 205 sequenced Pronkia specimens, including voucher specimen photograph(s), collection locality, date, habitat details and sequences, is available as a public dataset available at dx.doi.org/10.5883/DS-PRONKIA. Sequences are also available on GenBank and their accession numbers, voucher details and provenances are given in Suppl. material 1.

## Supplementary materials

Supplementary material 1 Taxon sampling and voucher information including specimen metadata, taxonomic classifications, collection details Data type docx https://binary.pensoft.net/file/1807641This dataset is made available under the Open Database License (http://opendatacommons.org/licenses/odbl/1.0/). The Open Database License (ODbL) is a license agreement intended to allow users to freely share, modify, and use this Dataset while maintaining this same freedom for others, provided that the original source and author(s) are credited. Donald L. J. Quicke, Darren F. Ward, Michael J. Sharkey, Buntika A. Butcher

Supplementary material 2 Ultrafast bootstrap (UFB) tree (1000 replicates) showing branch support values Data type pdf https://binary.pensoft.net/file/1807642This dataset is made available under the Open Database License (http://opendatacommons.org/licenses/odbl/1.0/). The Open Database License (ODbL) is a license agreement intended to allow users to freely share, modify, and use this Dataset while maintaining this same freedom for others, provided that the original source and author(s) are credited. Donald L. J. Quicke, Darren F. Ward, Michael J. Sharkey, Buntika A. Butcher

Supplementary material 3 Shimodaira-Hasegawa approximate likelihood ratio test (SH-aLRT) tree (1000 replicates) showing branch support values Data type pdf https://binary.pensoft.net/file/1807643This dataset is made available under the Open Database License (http://opendatacommons.org/licenses/odbl/1.0/). The Open Database License (ODbL) is a license agreement intended to allow users to freely share, modify, and use this Dataset while maintaining this same freedom for others, provided that the original source and author(s) are credited. Donald L. J. Quicke, Darren F. Ward, Michael J. Sharkey, Buntika A. Butcher

Supplementary material 4 Aligned COI sequences Data type txt https://binary.pensoft.net/file/1807644This dataset is made available under the Open Database License (http://opendatacommons.org/licenses/odbl/1.0/). The Open Database License (ODbL) is a license agreement intended to allow users to freely share, modify, and use this Dataset while maintaining this same freedom for others, provided that the original source and author(s) are credited. Donald L. J. Quicke, Darren F. Ward, Michael J. Sharkey, Buntika A. Butcher

Supplementary material 5 Aligned elongation factor-1 alpha (EF1-α) sequences Data type fas https://binary.pensoft.net/file/1807655This dataset is made available under the Open Database License (http://opendatacommons.org/licenses/odbl/1.0/). The Open Database License (ODbL) is a license agreement intended to allow users to freely share, modify, and use this Dataset while maintaining this same freedom for others, provided that the original source and author(s) are credited. Donald L. J. Quicke, Darren F. Ward, Michael J. Sharkey, Buntika A. Butcher

Supplementary material 6 Aligned 18S sequences; secondary structure interpretations and pairing and non-pairing bases indicated by “p” and “u”, respectively Data type txt https://binary.pensoft.net/file/1807646This dataset is made available under the Open Database License (http://opendatacommons.org/licenses/odbl/1.0/). The Open Database License (ODbL) is a license agreement intended to allow users to freely share, modify, and use this Dataset while maintaining this same freedom for others, provided that the original source and author(s) are credited. Donald L. J. Quicke, Darren F. Ward, Michael J. Sharkey, Buntika A. Butcher

Supplementary material 7 Aligned 28S sequences; secondary structure interpretations and pairing and non-pairing bases indicated by “P” and “U”, respectively Data type txt https://binary.pensoft.net/file/1807648This dataset is made available under the Open Database License (http://opendatacommons.org/licenses/odbl/1.0/). The Open Database License (ODbL) is a license agreement intended to allow users to freely share, modify, and use this Dataset while maintaining this same freedom for others, provided that the original source and author(s) are credited. Donald L. J. Quicke, Darren F. Ward, Michael J. Sharkey, Buntika A. Butcher

Supplementary material 8 Aligned 16S sequences; secondary structure interpretations and pairing and non-pairing bases indicated by “p” and “u”, respectively Data type txt https://binary.pensoft.net/file/1807650This dataset is made available under the Open Database License (http://opendatacommons.org/licenses/odbl/1.0/). The Open Database License (ODbL) is a license agreement intended to allow users to freely share, modify, and use this Dataset while maintaining this same freedom for others, provided that the original source and author(s) are credited. Donald L. J. Quicke, Darren F. Ward, Michael J. Sharkey, Buntika A. Butcher

Supplementary material 9 DNA sequence matrix analysed in fasta format with the order of sequences being Data type fas Explanation note COI (665 bases); EF1𝛼 (418 bases); 18S pairing bases (797 bases); 18S unpairing bases (549 bases); 28S pairing bases (453 bases); 28S unpairing bases (182 bases); 16S pairing bases (311 bases); 16S unpairing bases (224 bases). https://binary.pensoft.net/file/1807654This dataset is made available under the Open Database License (http://opendatacommons.org/licenses/odbl/1.0/). The Open Database License (ODbL) is a license agreement intended to allow users to freely share, modify, and use this Dataset while maintaining this same freedom for others, provided that the original source and author(s) are credited. Donald L. J. Quicke, Darren F. Ward, Michael J. Sharkey, Buntika A. Butcher
