# Supplementary material for: Revised relationships of the endemic New Zealand subfamily Pronkiinae stat. nov. (Hymenoptera, Braconidae)
Source: Zookeys. 2026 Oct 13;1294:313–31. doi: 10.3897/zookeys.1294.210518 (PMC13684312; doi:10.3897/zookeys.1294.210518)
Supplement: Supplementary file 1 [file zookeys-1294-313_article-210518__-s002.pdf]

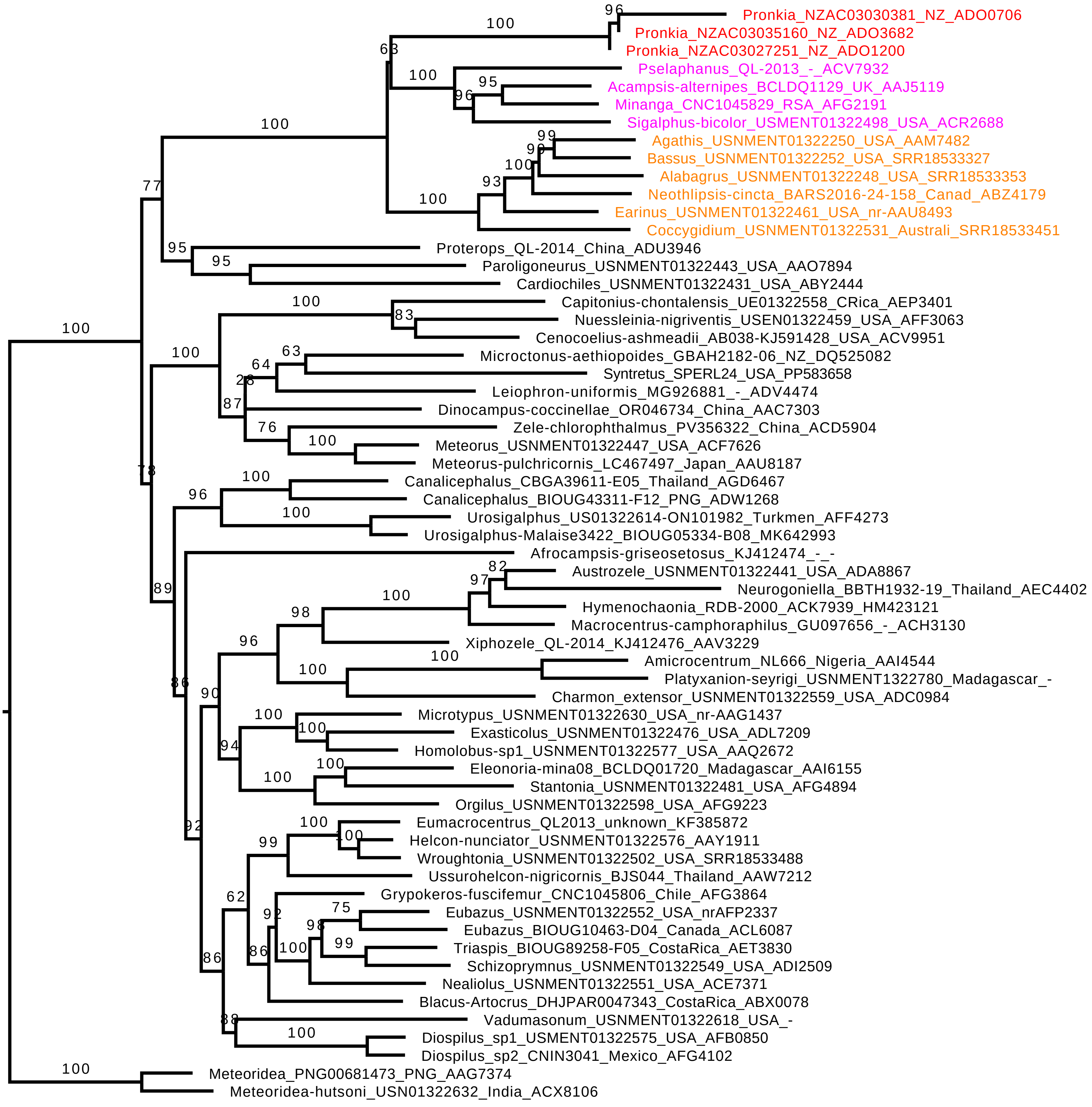
Pronkia_NZAC03030381_NZ_ADO0706
Pronkia_NZAC03035160_NZ_ADO3682
Pronkia_NZAC03027251_NZ_ADO1200
Pselaphanus_QL-2013_-_ACV7932
Acampsis-alternipes_BCLDQ1129_UK_AAJ5119
Minanga_CNC1045829_RSA_AFG2191
Sigalphus-bicolor_USMENT01322498_USA_ACR2688
Agathis_USNMENT01322250_USA_AAM7482
Bassus_USNMENT01322252_USA_SRR18533327
Alabagrus_USNMENT01322248_USA_SRR18533353
Neothlipsis-cincta_BARS2016-24-158_Canad_ABZ4179
Earinus_USNMENT01322461_USA_nr-AAU8493
Coccygidium_USNMENT01322531_Australi_SRR18533451
Proterops_QL-2014_China_ADU3946
Paroligoneurus_USNMENT01322443_USA_AAO7894
Cardiochiles_USNMENT01322431_USA_ABY2444
Capitonius-chontalensis_UE01322558_CRica_AEP3401
Nuessleinia-nigriventis_USEN01322459_USA_AFF3063
Cenocoelius-ashmeadii_AB038-KJ591428_USA_ACV9951
Microctonus-aethiopoides_GBAH2182-06_NZ_DQ525082
Syntretus_SPERL24_USA_PP583658
Leiophron-uniformis_MG926881_-_ADV4474
Dinocampus-coccinellae_OR046734_China_AAC7303
Zele-chlorophthalmus_PV356322_China_ACD5904
Meteorus_USNMENT01322447_USA_ACF7626
Meteorus-pulchricornis_LC467497_Japan_AAU8187
Canalicephalus_CBGA39611-E05_Thailand_AGD6467
Canalicephalus_BIOUG43311-F12_PNG_ADW1268
Urosigalphus_US01322614-ON101982_Turkmen_AFF4273
Urosigalphus-Malaise3422_BIOUG05334-B08_MK642993
Afrocampsis-griseosetosus_KJ412474_-_-
Austrozele_USNMENT01322441_USA_ADA8867
Neurogoniella_BBTH1932-19_Thailand_AEC4402
Hymenochaonia_RDB-2000_ACK7939_HM423121
Macrocentrus-camphoraphilus_GU097656_-_ACH3130
Xiphozele_QL-2014_KJ412476_AAV3229
Amicrocentrum_NL666_Nigeria_AAI4544
Platyxanion-seyrigi_USNMENT1322780_Madagascar_-
Charmon_extensor_USNMENT01322559_USA_ADC0984
Microtypus_USNMENT01322630_USA_nr-AAG1437
Exasticolus_USNMENT01322476_USA_ADL7209
Homolobus-sp1_USNMENT01322577_USA_AAQ2672
Eleonoria-mina08_BCLDQ01720_Madagascar_AAI6155
Stantonia_USNMENT01322481_USA_AFG4894
Orgilus_USNMENT01322598_USA_AFG9223
Eumacrocentrus_QL2013_unknown_KF385872
Helcon-nunciator_USNMENT01322576_AAY1911
Wroughtonia_USNMENT01322502_USA_SRR18533488
Ussurohelcon-nigricornis_BJS044_Thailand_AAW7212
Grypokeros-fuscifemur_CNC1045806_Chile_AFG3864
Eubazus_USNMENT01322552_USA_nrAFP2337
Eubazus_BIOUG10463-D04_Canada_ACL6087
Triaspis_BIOUG89258-F05_CostaRica_AET3830
Schizoprymnus_USNMENT01322549_USA_ADI2509
Nealiolus_USNMENT01322551_USA_ACE7371
Blacus-Artocrus_DHJPAR0047343_CostaRica_ABX0078
Vadumasonum_USNMENT01322618_USA_-
Diospilus_sp1_USMENT01322575_USA_AFB0850
Diospilus_sp2_CNIN3041_Mexico_AFG4102
Meteoridea_PNG00681473_PNG_AAG7374
Meteoridea-hutsoni_USN01322632_India_ACX8106
