# Supplementary material for: Revised relationships of the endemic New Zealand subfamily Pronkiinae stat. nov. (Hymenoptera, Braconidae)
Source: Zookeys. 2026 Oct 13;1294:313–31. doi: 10.3897/zookeys.1294.210518 (PMC13684312; doi:10.3897/zookeys.1294.210518)
Supplement: Supplementary file 3 [file zookeys-1294-313_article-210518__-s001.docx]

Sequences used in the analyses.

| Subfamily | Genus | Species*, voucher code, provenance, GenBank accession, BIN (COI only or of other spp if known) | | | | |
| --- | --- | --- | --- | --- | --- | --- |
|  |  | COI | EF1-𝛂 | 18S | 28S | 16S |
| Pronkiinae stat. nov. | *Pronkia* sp. 1 | NZAC03030381,  New Zealand, ADO0706 | — | NZAC03030381,  New Zealand, ADO0706 | NZAC03030381,  New Zealand, ADO0706 | — |
| — | *Pronkia* sp. 2 | NZAC03035160, New Zealand,  ADO3682 | NZAC03035160, New Zealand | NZAC03035160, New Zealand,  ADO3682 | NZAC03035160, New Zealand,  ADO3682 | NZAC03035160,  New Zealand, |
| — | *Pronkia* sp. 3 | NZAC03027251, New Zealand,  ADO1200 | NZAC03027251, New Zealand | NZAC03027251, New Zealand,  ADO1200 | NZAC03027251, New Zealand,  ADO1200 | — |
| Agathidinae | *Agathis* spp | USNMENT01322250, USA, -, AAM7482 | CBG-A19161-H09, USA, pending, ADM42s | USNMENT01322249, SRR18533352 | sp2, NL-2005, DQ201889 | USNMENT01322249 |
| — | *Alabagrus* sp. 1 | USNMENT01322248, USA, SRR18533353 | — | USNMENT01322248, USA, SRR18533353 | USNMENT01322248, USA, SRR18533353 | USNMENT01322248, USA, SRR18533353 |
| — | *Bassus* sp. 1** | USNMENT01322252, USA, SRR18533327 | USNMENT01322252, USA, SRR18533327 | USNMENT01322252, USA, SRR18533327 | USNMENT01322252, USA, SRR18533327 | USNMENT01322252, USA, SRR18533327 |
|  | *Coccygidium* sp. 1 | USNMENT01322531, Australia, SRR18533451, - | — | USNMENT01322531, Australia, SRR18533451 | USNMENT01322531, Australia, SRR18533451 | USNMENT01322531, Australia, SRR18533451 |
| — | *Earinus* spp | sp. 1, USNMENT01322461, USA, SRR18533515, nr-AAU8493 | *limitaris*, OPPFA1208-17, pending, AAU8493 | sp. 1, USNMENT01322461, USA, SRR18533515 | sp. 1, USNMENT01322461, USA, SRR18533515 | sp. 1, USNMENT01322461, USA, SRR18533515 |
| — | *Neothlipsis* spp | *cincta*, BARS2016-24-158, Canada, pending, ABZ4179 | *cincta*, 367204, USA, pending, AAE1347 | *cincta*, USNMENT01322251, USA, SRR18533350 | sp. 1, H1285, USA, MF098347 | *cincta*, USNMENT01322251, USA, SRR18533350 |
| Sigalphinae | *Acampsis alternipes* | BCLDQ1129, UK, JF962457, AAJ5119 | -, UK, Z83674 | CNC1045828, Germany, SRR18432342 | -, -, MF098313 | CNC1045828, Germany, SRR18432342 |
| — | *Minanga* sp. 1 | CNC1045829, South Africa, -, AFG2191 | CNC1045829, South Africa, ON101898 | CNC1045829, South Africa, SRR18432341 | CNC1045829, South Africa, ON101898 | CNC1045829, South Africa, ON101898 |
| — | *Pselaphanus* spp | sp. 1, QL-2013, French Guiana, KF385876, ACV7932 | — | — | sp. 2, JM463, Brazil, EU427393 | sp. 1, QL-2013, French Guiana, KF385876 |
| — | *Sigalphus bicolor* | USMENT01322498, USA, SRR18533493, ACR2688 | — | USMENT01322498, USA, SRR18533493 | USMENT01322498, USA, SRR18533493 | USMENT01322498, USA, SRR18533493 |
| Meteorideinae | *Meteoridea hutsoni* | USNMENT01322632, India, SRR18533328, ACX8106 | — | USNMENT01322632, India, SRR18533328, | USNMENT01322632, India, SRR18533328, | USNMENT01322632, India, SRR18533328, |
| — | *Meteoridea* spp | sp. 1, PNG00681473, Papua New Guinea, pending, AAG7374 | — | — | sp. 2, RDB-1, Thailand, AJ416974 | sp. 1, PNG00681473, Papua New Guinea, MK642939 |
| Helconinae | *Helcon (Eumacrocentrus)* spp | sp, QL-2013, ?, USA, KF385872, - | *americanus*, BIOUG12469-F04, Canada, KR418350, AAY3477 | sp., USNMENT01322643, USA, SRR18533544 | *americanus*, BJS012, USA, JF979825-JF979688 | sp., QL-2013, USA, KF385872 |
| — | *Helcon (Helcon)* spp. | *nunciator*, USNMENT01322576, USA, -, AAY1911 | *nunciator*, USNENT01322576, USA, SRR18533403 | *nunciator*, USNMENT01322576, USA, SRR18533403 | *texanus*, JF979691-JF979828 | *nunciator*, USNMENT01322576, USA, SRR18533403 |
| — | *Helcon (Wroughtonia)* spp | sp. 1, USNMENT01322502, USA, SRR18533488, - | *ligator*, DM325, JQ519636 | sp. 1, USNMENT01322502, USA, SRR18533488 | sp. 1, USNMENT01322502, USA, SRR18533488 | sp. 1, USNMENT01322502, USA, SRR18533488 |
| — | *Ussurohelcon* spp. | *nigricornis*, BJS044, Thailand, JF963932, AAW7212 | sp., CBG-A40720-F11, Thailand, pending, AGF2730 | *nigricornis*, BJS044, Thailand, JF979572-AJ307445 | *nigricornis*, BJS044, Thailand, JF979711 JF979846 | — |
| Brachistinae | *Blacus (Artocrus)* spp | malaise0047, DHJPAR0047343, Costa Rica, pending, ABX0078 | malaise0047, DHJPAR0047343, Costa Rica, pending, | sp. 1, CNC1045873, Costa Rica, SRR18432334 | malaise0047, DHJPAR0047343, Costa Rica, pending, | sp. 1, CNC1045873, Costa Rica, SRR18432334 |
| — | *Diospilus* sp. 1 | USMENT01322575, USA, SRR18533404, AFB0850 | USNMENT01322575, USA, SRR18533404 | USNMENT01322575, USA, SRR18533404 | USNMENT01322575, USA, SRR18533404 | USNMENT01322575, USA, SRR18533404 |
| — | *Diospilus* spp | sp. 2, CNIN3041, Mexico, ON101862, AFG4102 | sp. 3, CBG-A35832-A07, Costa Rica, pending, AGB6196 | sp. 4, JS10-00005, Ecuador, KJ591201 | sp. 5, CHIP019-12, Canada, MG440040, AAB5730 | sp. 2 CNIN3041, Mexico, SRR18306452 |
| — | *Eubazus* spp. | sp. 1, USNMENT01322552, USA, SRR18533432, nr AFP2337 | *semirugosus*, -, Z83673, Switzerland, - | sp. 1, USNMENT01322552, USA, SRR18533432 | sp. 1, USNMENT01322552, USA, SRR18533432 | USNMENT01322552, USA, SRR18533432 |
| — | *Eubazus* spp. | sp. 2, BIOUG10463-D04, Canada, pending, ACL6087 | *semirugosus*, -, Switzerland, Z83673 | — | *semirugosus*, -, Switzerland, Z83608 | *semirugosus* -, Switzerland, AF176058 |
| — | *Grypokeros* spp. | *fuscifemur*, CNC1045806, Chile, SRR18432308, AFG3864 | — | *fuscifemur*, CNC1045806, Chile, SRR18432308 | BJS-20011 sp. 1, BJS112, Chile, JF97973 | *fuscifemur*, CNC1045806, Chile, SRR18432308 |
| — | *Nealiolus* sp. 1 | USNMENT01322551, USA, SRR18533433, ACE7371 | USNMENT01322551, USA, SRR18533433 | USNMENT01322551, USA, SRR18533433 | USNMENT01322551, USA, SRR18533433 | USNMENT01322551, USA, SRR18533433 |
| — | *Schizoprymnus* sp. 1 | USNMENT01322549, USA, SRR18533435, ADI2509 | — | USNMENT01322549, USA, SRR18533435 | USNMENT01322549, USA, SRR18533435 | USNMENT01322549 USA, SRR18533435 |
| — | *Triaspis* spp. | Malaise3830, BIOUG89258-F05, Costa Rica, pending, AET3830 | Malaise3830, BIOUG89258-F05, Costa Rica, pending, AET3830 | sp. 1, USNMENT01322475, USA, SRR18533458 | Malaise3830, BIOUG89258-F05, Costa Rica, pending, AET3830 | sp. 1, USNMENT01322475, USA, SRR18533458 |
| — | *Vadumasonum* sp. 1 | USNMENT01322618, USA, SRR18533339, - | — | USNMENT01322618, USA, SRR18533339 | USNMENT01322618, USA, SRR18533339 | USNMENT01322618, USA, SRR18533339 |
| Microtypinae | *Microtypus* spp | sp. 1, USNMENT01322630, USA, -, nr-AAG1437 | sp. 2, CBG-A15000-B11, Canada, pending, AAG1437 | *wesmaelii*, CNC1045824, SRR17486629 | *wesmaelii*, -, -, AJ302822, - | sp. 1, USNMENT01322630, USA, SRR18533329 |
| Amicrocentrinae | *Amicrocentrum* spp | sp., NL666, Nigeria, JF962898, AAI4544 | — | *concolor*, JS276, Malawi, JF962897 | *concolor*, JS276, Malawi, JF962897-JF9F962898 | — |
|  | *Platyxanion seyrigi* | USNMENT1322780, Madagascar, SRR25867742, - | — | — | USNMENT1322780, Madagascar, SRR25867742 | USNMENT1322780, Madagascar, SRR25867742 |
| Macrocentrinae | *Austrozele* spp | sp. 1, USNMENT01322441, USA, SRR18533303, ADA8867 | *jorgesoberoni*, DHJPAR0042749, Costa Rica, pending, AAF0520 | sp. 1, USNMENT01322441, USA, SRR18533303 | sp. 1, USNMENT01322441, USA, SRR18533303 | sp.1, USNMENT01322441, USA, SRR18533303 |
| — | *Hymenochaonia* spp | sp. 1, RDB-2000, Costa Rica, HM423121, ACK7939 | sp. 2, USNMENT01322584, USA, SRR18533395 | sp. 2, USNMENT01322584, USA, SRR18533395 | sp. 1, RDB-2000, Costa Rica, AJ302817 | sp. 2, USNMENT01322584, USA, SRR18533395 |
| — | *Macrocentrus* spp | *camphoraphilus*, ?, GU097656, ?China, ACH3130 | *michaelstroudi*, DHJPAR0058830, Costa Rica, pending, ACK7466 | *melanogaster*, ?, ?China, AY920274 | sp. 1, USNMENT01322585, USA, SRR18533394 | *camphoraphilus*, ?, ?China,GU097656 |
| — | *Neurogoniella* sp | CCDB-30084-G6, Thailand, pending, AEC4402 | CCDB-30084-G6, Thailand, pending | — | — | — |
| Charmontinae | *Charmon* spp | *extensor*, USNMENT01322559, USA, ADC0984 | sp. 1, USNMENT01322560, USA, SRR18533421 | *rufithorax*, ?, ?China, AY920272 | sp. 1, USNMENT01322560, USA, SRR18533421 | sp. 1, USNMENT01322560, USA, SRR18533421 |
| Homolobinae | *Exasticolus* spp | sp. 1, USNMENT01322476, USA, SRR18533457, ADL7209 | sp. 2, CCDB-31606-C06, Costa Rica, pending, AGD8738 | sp. 1, USNMENT01322476, USA, SRR18533457 | sp. 3, MS-2002, Panama, AJ517426 | — |
| — | *Homolobus* spp | sp. 1, USNMENT01322577, USA, AAQ2672 | *truncator*, 09BBHYM-271, USA, pending, AAZ4462 | *fuscinervis*, JS10-00063, Ecuador, KJ591202 | *fuscinervis*, JS10-00063, Ecuador, KJ591342 | sp. 1, USNMENT01322577, USA |
| Xiphozelinae | *Xiphozele* spp | sp. 1, QL-2014, ?China, KJ412476, AAV3229 | — | sp. 2, ZOO35, Thailand, JF979607 | sp. 3, RDB-2000, Indonesia (Java), AJ302931-AJ307461 | sp. 1, QL-2014, ?China,KJ412476 |
| Cenocoeliinae | *Capitonius* spp | *chontalensis*, UE01322558, Costa Rica, pending, AEP3401 | *chontalensis*, UE01322558, Costa Rica, pending, AEP3401 | *chontalensis*, UE01322558, Costa Rica, SRR18533425 | *chontalensis*, KP011, Costa Rica, JF979767-JF979902, - | sp. 1, QL-2013, USA, KF385869 |
| — | *Nuessleinia nigriventis* | USNMENT01322459, USA, SRR18533518, AFF3063 | — | USMENT01322459, USA, SRR18533518 | USMENT01322459, USA, SRR18533518 | USMENT01322459, USA, SRR18533518 |
| — | *Cenocoelius* spp | *ashmeadii*, AB038, USA, KJ591428, ACV9951 | sp. 1, CCDB-07373 H11, French Guiana, AAV6259 | *ashmeadii*, USNMENT01322645, USA, SRR18533542 | *ashmeadii*, USNMENT01322645, USA, SRR18533542 | *ashmeadii*, USNMENT01322645, USA, SRR18533542 |
| Euphorinae | *Dinocampus-coccinellae* | -, OR046734, China, AAC7303 | — | JS10_00209, Sweden, KJ591073 | -, UK, AJ302807 | -, ?Brazil, MG253265, - |
| — | *Leiophron-*spp | *uniformis*, MG926881, -, ADV4474 | — | *uniformis*, SAMN26554234, SRR18533506 | *deficiens*, JS01000540, MG913709 | *uniformis*, AF176057 |
| — | *Meteorus* sp 1 | sp. 1, USNMENT01322447, USA, SRR18533524, ACF7626 | sp. 1, USNMENT01322447, USA, SRR18533524 | sp. 1, USNMENT01322447, USA, SRR18533524 | sp. 1, USNMENT01322447, USA, SRR18533524 | sp. 1, USNMENT01322447, USA, SRR18533524 |
| — | *Meteorus* spp | *pulchricornis* , LC467497, Japan, AAU8187 | *arcticus*, CHARS00409-A10, ACK3223 | aff. *versicolor*, JS10-00110, KJ591097 | *pulchricornis*, HQ263734 | *pulchricornis,* NC-053259 |
| — | *Microctonus* spp | *aethiopoides*, GBAH2182-06, NZ, DQ525082 | — | sp. 2, USNMENT01322570, USA, SRR18533408 | *aethiopoides*, haplotype-1-28, AY129823 | *aethiopoides*, SI3d1  , France, EU078380 |
| — | *Syntretus* spp | sp. 1, SPERL24, USA, PP583658 | — | sp. 2, USNMENT01322572, USA, SRR18533406 | *zuijleni*, JS10, ,00156, -6, KJ591324 | sp. 1, SPERL24, USA, PP583658 |
| — | *Zele* spp | *chlorophthalmus*, -, China, PV356322, ACD5904 | — | *deceptor*, -, ?, AY920273 | *chlorophthalmus*, DNA_44, Sweden, HQ263762 | *chlorophthalmus*, -, ?China, NC_039181 |
| Orgilinae | *Eleonoria mina08* | BCLDQ01720, Madagascar, AAI6155 | — | — | EU427385 | — |
| — | *Orgilus* sp. 1 | USNMENT01322598, USA, SRR18533382, AFG9223 | USNMENT01322598, USA, SRR18533382, | USNMENT01322598, USA, SRR18533382 | USNMENT01322598, USA, SRR18533382 | USNMENT01322598, USA, SRR18533382 |
| — | *Stantonia* spp | sp. 1, USNMENT01322481, USA, SRR18533455, AFG4894 | sp. 2, CCDB-23210-A06, PNG, pending | sp. 1, USNMENT01322481, USA, SRR185334551 | sp. 1, USNMENT01322481, USA, SRR18533455 | sp. 1, USNMENT01322481, USA, SRR18533455 |
| Afrocampsinae | *Afrocampsis griseosetosus* | -, ?, Republic of Congo, KJ412474 | — | CNC1045799, South Africa, SRR18432357 | -, ?, Republic of Congo, AJ517428 | -, ?, Republic of Congo, KJ412474 |
| — | *Canalicephalus* spp | sp. 1, CBGA39611-E05, Thailand, pending, AGD6467 | sp. 1, CBGA39611-E05, Thailand, pending, | — | sp. 2, RDB-2000, Malaysia (Sabah), AJ302799 | sp. 1, CBGA39611-E05, Thailand, pending |
| — | *Canalicephalus* sp. 3 | sp. 3, BIOUG43311-F12, Papua New Guinea, pending, ADW1268 | — | — | — | sp. 3, BIOUG43311-F12, Papua New Guinea, pending, ADW1268 |
| — | *Urosigalphus* sp. 1 | sp. 1, ON101982, Turkmenistan (introduced), AFF4273 | sp. 1, USNMENT01322614, USA, SRR18533343 | sp. 1, USNMENT01322614, USA, SRR18533343 | sp. 1, USNMENT01322614, USA, SRR18533343 | sp. 1, USNMENT01322614, USA, SRR18533343 |
| — | *Urosigalphus* spp | Malaise3422, BIOUG05334-B08, Costa Rica, MK642993 | Malaise3422, BIOUG05334-B08, Costa Rica, MK722127 | — | sp. 2, RDB-2000, ex culture Texas A&M University, AJ302923 | Malaise3422, BIOUG05334-B08, Costa Rica, MK642931 |
| Proteropinae | *Proterops* spp | sp. 1, QL-2014, ?China, KJ412477, ADU3946 | *vickifunkae*, ASHYC4688-10, Costa Rica, DHJPAR0037943, HQ548632, AAM1715 | cf. *proteroptoides*, USNMENT01322650, USA, SRR18533537 | cf. *proteroptoides*, USNMENT01322650, USA, SRR18533537 | cf. *proteroptoides*, USNMENT01322650, USA, SRR18533537 |
| Ichneutinae | *Paroligoneurus* spp | sp. 1, USNMENT01322443, USA, SRR18533552, AAO7894 | sp. 2, JCB-2006, -, DQ538689, - | sp. 1, USNMENT01322443, USA, SRR18533552 | sp. 1, USNMENT01322443, USA, SRR18533552 | sp. 1, USNMENT01322443, USA, SRR18533552 |
| Cardiochilinae | *Cardiochiles* spp | sp. 1, USNMENT01322431, USA, SRR18533314, ABY2444 | sp. 2 *apicalis* group, CNC924605, USA, SRR18432325 | sp. 1, USNMENT01322431, USA, SRR18533314 | sp. 1, USNMENT01322431, USA, SRR18533314 | sp. 1, USNMENT01322431, USA, SRR18533314 |

* if all the same

** BOLD ID engine (https:// https://id.boldsystems.org/) identifies the assembled COI sequence as 98.78% similar to *Aerophilus binominatus* (BOLD:AAA7526)
